# Retina-specific loss of *Ikbkap/Elp1* causes mitochondrial dysfunction that leads to selective retinal ganglion cell degeneration in a mouse model of familial dysautonomia

**DOI:** 10.1242/dmm.033746

**Published:** 2018-07-30

**Authors:** Yumi Ueki, Veronika Shchepetkina, Frances Lefcort

**Affiliations:** Department of Cell Biology and Neuroscience, Montana State University, Bozeman, MT 59717, USA

**Keywords:** IKAP/ELP1, Familial dysautonomia, Mitochondria, Retinal degeneration, Retinal ganglion cells

## Abstract

Familial dysautonomia (FD) is an autosomal recessive disorder marked by developmental and progressive neuropathies. It is caused by an intronic point-mutation in the *IKBKAP/ELP1* gene, which encodes the inhibitor of κB kinase complex-associated protein (IKAP, also called ELP1), a component of the elongator complex. Owing to variation in tissue-specific splicing, the mutation primarily affects the nervous system. One of the most debilitating hallmarks of FD that affects patients' quality of life is progressive blindness. To determine the pathophysiological mechanisms that are triggered by the absence of IKAP in the retina, we generated retina-specific *Ikbkap* conditional knockout (CKO) mice using *Pax6-Cre*, which abolished *Ikbkap* expression in all cell types of the retina. Although sensory and autonomic neuropathies in FD are known to be developmental in origin, the loss of IKAP in the retina did not affect its development, demonstrating that IKAP is not required for retinal development. The loss of IKAP caused progressive degeneration of retinal ganglion cells (RGCs) by 1 month of age. Mitochondrial membrane integrity was breached in RGCs, and later in other retinal neurons. In *Ikbkap* CKO retinas, mitochondria were depolarized, and complex I function and ATP were significantly reduced. Although mitochondrial impairment was detected in all *Ikbkap*-deficient retinal neurons, RGCs were the only cell type to degenerate; the survival of other retinal neurons was unaffected. This retina-specific FD model is a useful *in vivo* model for testing potential therapeutics for mitigating blindness in FD. Moreover, our data indicate that RGCs and mitochondria are promising targets.

## Abstract (precis)

**Summary:** The elongator subunit IKBKAP/ELP1 is not required for development, but is essential for maintaining mitochondrial function and retina morphology. Loss of this subunit causes progressive, selective degeneration of retinal ganglion cells.

## INTRODUCTION

Familial dysautonomia (FD; MIM223900) is an autosomal recessive congenital neuropathy that is caused by an intronic mutation in the *IKBKAP* gene, which encodes the inhibitor of κB kinase complex-associated protein (IKAP), also called elongator complex protein 1 (ELP1) (Anderson et al., 2001; Dong et al., 2002; Riley et al., 1949; Slaugenhaupt et al., 2001). A point-mutation in intron 20 results in tissue-specific exon skipping and generates an unstable mRNA, causing a loss-of-function phenotype predominantly in the nervous system (Cuajungco et al., 2003; Dietrich et al., 2012; Keren et al., 2010). FD patients suffer from congenital and progressive neuropathies, including reduced peripheral afferent sensory function, unstable blood pressure, hypotonia, poor growth and spinal curvature; patients often die in early adulthood owing to sudden unexpected death during sleep (Axelrod, 2002; Palma et al., 2014, 2017; Riley et al., 1949). The complete functional repertoire of IKAP/ELP1 remains unresolved, but includes a key role as the scaffolding subunit of the six-subunit elongator complex (ELP1-6) that modifies wobble uridine subunits of tRNA during translation (Bauer and Hermand, 2012; Chen et al., 2009a; Huang et al., 2005). In its absence, translation of codon-biased mRNAs is impaired, resulting in perturbations in levels of specific proteins (Goffena et al., 2018). Either as a direct or indirect consequence of this altered translation, *Ikbkap* conditional knockout (CKO) neurons exhibit impaired axonal transport, target innervation and cell survival (Abashidze et al., 2014; Chaverra et al., 2017; Close et al., 2006; George et al., 2013; Hunnicutt et al., 2012; Jackson et al., 2014; Johansen et al., 2008; Lefler et al., 2015; Naftelberg et al., 2016; Naumanen et al., 2008; Tourtellotte, 2016; Ueki et al., 2016).

FD is classified as a hereditary sensory and autonomic neuropathy (HSAN III), yet closer examination of both the patient and mouse-model phenotypes reveals central nervous system (CNS) pathology (Axelrod et al., 2010; Mendoza-Santiesteban et al., 2014; Ochoa, 2003). Furthermore, accumulating evidence demonstrates a vital role of the elongator complex in the CNS: variants in *ELP2* are associated with neurodevelopmental disability (Cohen et al., 2015; Franić et al., 2015) and variants in *ELP3* with amyotrophic lateral sclerosis (ALS) (Simpson et al., 2009). In addition, loss of *ELP4* causes Rolandic epilepsy syndrome, autism and intellectual disability (Addis et al., 2015; Gkampeta et al., 2014; Nguyen et al., 2010; Reinthaler et al., 2014; Strug et al., 2009). One of the major clinical hallmarks of FD is progressive blindness, which starts at an early age as a result of the progressive loss of retinal ganglion cells (RGCs) (Mendoza-Santiesteban et al., 2014, 2012, 2017). Patients are often legally blind by their thirties. In the FD community, there is mounting interest in developing treatments to ameliorate the blindness by preventing the progressive RGC loss in order to improve the quality of life of FD patients.

The pathophysiological mechanisms underlying the loss of vision have not been the focus of any study until recently, however. Our recent work with *Ikbkap* CKO mice, which lack *Ikbkap* in both CNS and peripheral nervous system (PNS) neurons, demonstrated that loss of *Ikbkap* in RGCs causes their progressive death (Ueki et al., 2016), recapitulating the retinal phenotype of the FD patients (Mendoza-Santiesteban et al., 2014, 2017). Unfortunately, with this model we were unable to analyze the consequence of *Ikbkap* loss in cell types other than RGCs, as the *Ikbkap* deletion was primarily restricted to RGCs in the retina. Moreover, *Ikbkap* was deleted in the majority of cell types in both the PNS and CNS, so we were unable to determine whether the loss of RGCs was the direct or indirect consequence of loss of *Ikbkap* throughout the nervous system. These mice had a severe progressive peripheral neuropathy and CNS impairments (Chaverra et al., 2017). To overcome these complications, in this study we have generated and characterized a new mouse FD retina model by conditional deletion of *Ikbkap* solely in the retina, using a retina-specific *Pax6-Cre*, which is expressed in the retinal progenitors; thus, all retinal cell types are affected (Marquardt et al., 2001). This retina-specific FD model system allowed us to directly interrogate the consequence of loss of *Ikbkap* in all retinal cell types in the context of an otherwise healthy nervous system. Our data demonstrate that loss of *Ikbkap* solely in the retina causes RGC degeneration, yet the survival of other retinal cell types is unaffected. In addition, there was no developmental phenotype in the FD retinas, establishing for the first time that the loss of RGCs occurs postnatally and is a progressive neurodegeneration as in the human disease.

Defects in mitochondria have been implicated in many, if not all, neurodegenerative disorders (Schon and Przedborski, 2011). Similar to FD, two other optic neuropathies that are considered mitochondrial diseases, Leber's hereditary optic neuropathy (LHON) and dominant optic atrophy (DOA), are also characterized by loss of vision owing to slow, progressive RGC degeneration (Carelli et al., 2009; Chevrollier et al., 2008; Kirches, 2011; Newman and Biousse, 2004; Yu-Wai-Man et al., 2011; Zanna et al., 2008). In this study, we hypothesized that the progressive demise of retinal neurons in FD results from mitochondrial dysfunction. Our data demonstrate perturbation of mitochondrial membrane integrity and function, as well as decreased ATP content in the *Ikbkap* CKO retinas. Interestingly, although mitochondria of all *Ikbkap*-deficient retinal neurons seem to be affected, RGCs are the only cell type that degenerate. Together, these results suggest that RGCs and mitochondria are promising targets for future therapeutics to mitigate the progressive loss of vision in FD patients.

## RESULTS

### IKAP expression is significantly reduced in the *Ikbkap* CKO retina

We previously generated *Ikbkap* CKO mice using a *TUBA1a* promoter-driven Cre (*Tuba1α-Cre*), which targets ∼90% of postmitotic RGCs but not other cell types in the retina (Ueki et al., 2016). We demonstrated that loss of IKAP in the RGCs caused a slow, progressive RGC degeneration most severely in the temporal retina, later followed by indirect photoreceptor loss and complete retinal disorganization. In this model, however, *Ikbkap* was deleted from the majority of CNS neurons and approximately 40% of PNS neurons; thus, whether all or some of these phenotypes were directly due to autonomous loss of *Ikbkap* in retinal cells could not be determined. In this current study, we have generated a retinal-specific *Ikbkap* CKO mouse using *Pax6*-*Cre*. *Pax6-Cre* is expressed in retinal progenitors, affecting all six types of retinal neurons and Müller glia, and its expression is restricted solely to the retina (Marquardt et al., 2001). Therefore, we can characterize the effect of autonomous loss of *Ikbkap* not only in RGCs, but in all retinal neurons. To identify appropriate therapeutic targets, it is important to understand the consequence of IKAP loss in all retinal neurons and not only in RGCs.

**Fig. 1.**
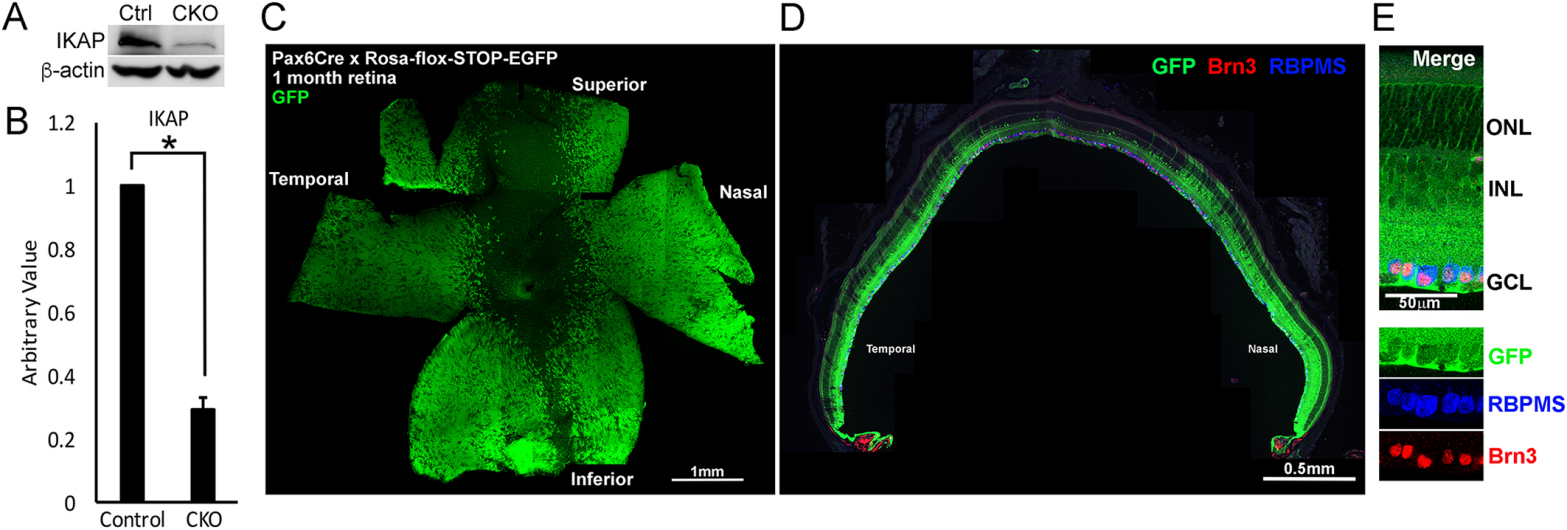
**Generation of a retinal model of FD.** *Pax6-Cre^+^;Ikbkap^Flox/Flox^* mice were generated to delete *Ikbkap* selectively from the retina. (A) Representative IKAP western blot at 1 month. (B) *Pax6-Cre^+^;Ikbkap^Flox/Flox^* (CKO) retinas show a 70% decrease in IKAP expression compared with *Pax6-Cre^−^;Ikbkap^Flox/Flox^* (Control; Ctrl) retinas at 1 month. *P=0.000016 with Student's *t*-test (*n*=5). (C-E) *Pax6-Cre* expression was analyzed on retinal (C) flatmounts and (D) cross-sections using *Rosa-EGFP Cre* reporter mice at 1 month. *Cre* expression was detected in the mid-peripheral retinas and, typically, 50-85% of the retina was affected in each eye. Images in C and D are composites. Multiple images were taken and reconstructed to show whole retinal (C) flatmount and (D) cross sections. (E) Representative images of retinal cross-sections in the *Cre*-expressing region are shown. All cell types of the retina expressed *Cre* (GFP^+^), including RGCs (Brn3^+^ and/or RBPMS^+^). GCL, ganglion cell layer; INL, inner nuclear layer; ONL, outer nuclear layer.

Western blot analysis showed a significant reduction in IKAP protein in the *Pax6-Cre Ikbkap* CKO retinas at 1 month compared with littermate control retinas (Fig. 1A); on average, there was over 70% reduction in IKAP expression in the CKO retina (Fig. 1B). Previous studies show that *Pax6-Cre* is expressed in the peripheral retina, but not in the central retina (Georgi and Reh, 2010; La Torre et al., 2013; Marquardt et al., 2001). We also observed this peripheral bias when we crossed *Pax6-Cre* mice with *Rosa-EGFP Cre* reporter mice (Fig. 1C,D). In a typical *Cre^+^* retina, 50-85% retinal regions were GFP^+^ (Fig. 1C), which corresponds with ∼70% reduction in IKAP expression (Fig. 1A,B). When we analyzed *Cre* reporter retinal cross-sections at 1 month, all the retinal cell types including RGCs [identified using the retinal markers Brn transcription factor (Brn3^+^) and/or RNA-binding protein with multiple splicing (RBPMS^+^)] expressed GFP (Fig. 1E), as reported in previous studies (Marquardt et al., 2001). We used *Pax6-Cre Ikbkap* CKO that had been crossed with the *Rosa-EGFP Cre* reporter strain for immunohistological experiments, in order to identify the retinal regions that were deficient in IKAP. In summary, we have established a useful mouse model of FD blindness.

### Loss of IKAP induces progressive RGC degeneration, whereas other retinal cell types are unaffected

**Fig. 2.**
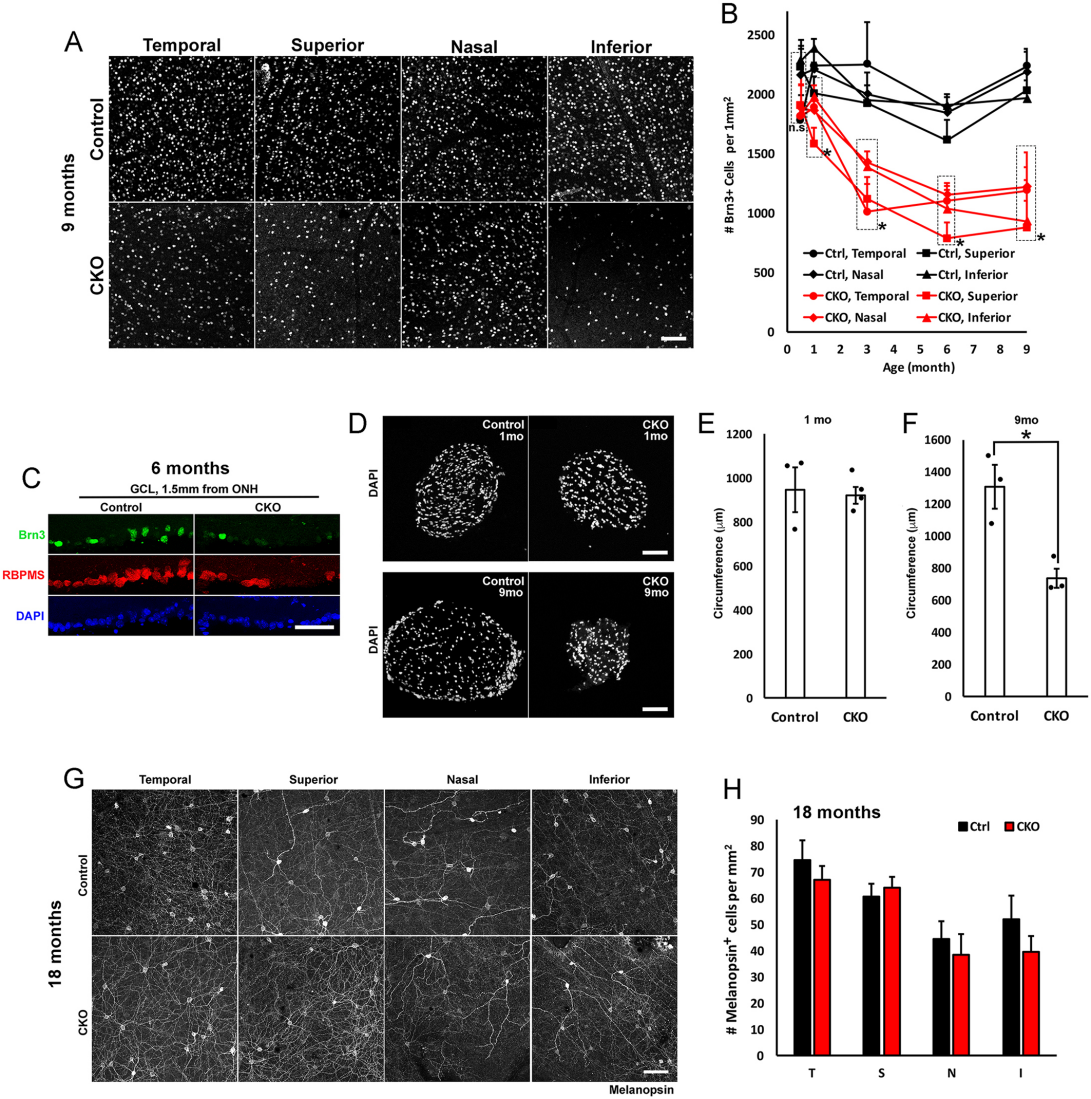
**Loss of IKAP in the retina causes progressive loss of RGCs.** (A) At 9 months, *Pax6-Cre Ikbkap* CKO retinas show a decrease in the number of Brn3^+^ RGCs at 1 mm from the optic nerve. (B) Significant loss of Brn3^+^ RGCs in CKO retinas was detected in all quadrants (temporal, superior, nasal and inferior) as early as 1 month, and the loss was progressive. Rapid RGC loss occurs between 1 month and 3 months. There was no significant difference in the number of RGCs at P14, when retinal development has completed. P14 (*n*=4), 1 month (*n*=6), 3 months (*n*=4), 6 months (*n*=6) and 9 months (*n*=3). The number of RGCs in the control and CKO retinas at the same region and age were compared with Student's *t*-test (*P<0.05). (C) Cross-sections of 6 month temporal retinas at 1.5 mm from the optic nerve. There was an apparent reduction in the number of Brn3^+^ RGCs and total RGCs (RBPMS^+^, pan-RGC marker). (D) Cross-sections of optic nerves at 1 month (top) and 9 months (bottom) were visualized with DAPI staining. (E) There was no difference in control and CKO optic nerve circumference at 1 month. *P*=0.41 (not significant) with Student's *t*-test (*n*=3). (F) By 9 months, there is a 45% decrease in CKO optic nerve circumference compared with controls. *P=0.018 with Student's *t*-test (*n*=3). (G,H) There was no loss of melanopsin^+^ intrinsically photosensitive RGCs at 18 months. Melanopsin^+^ cells were counted at 1 mm from the optic nerve in each quadrant. *P*=0.2 (temporal), *P*=0.3 (superior), *P*=0.3 (nasal) and *P*=0.1 (inferior) with Student's *t*-test (*n*=5). Scale bars: 100 µm (A,D,G) and 50 µm (C).

As expected from our previous study (Ueki et al., 2016), we observed progressive loss of RGCs owing to loss of IKAP in the retina. Staining of 9 month retinal flatmounts and 6 month retinal cross-sections with RGC markers Brn3 (Fig. 2A) and RBPMS (Fig. 2C), respectively, showed an apparent reduction in the RGC number in all four retinal quadrants. Quantification of RGC number reveals a significant loss of RGCs at 1 month, followed by a rapid reduction in the RGC number over the subsequent 2 months (Fig. 2B). By 6 months, there was a 40-60% loss of RGCs in CKO retinas compared with controls. This progressive loss of RGCs is not due to *Pax6-Cre* expression itself or a result of the loss of one *Ikbkap* allele, as there was no significant difference in RGC counts in *Pax6-Cre^+^; Ikbkap^Flox/+^* or *Pax6-Cre^−^; Ikbkap^Flox/Flox^* (control) retinas at 18 months (Fig. S1). To determine whether IKAP is required in the developing retina, we quantified RGC number at postnatal day 14 (P14), the age when retinal development has completed. Importantly, there was no significant difference in RGC number between control and CKO retinas at P14, indicating that RGC development does not require IKAP (Fig. 2B). Thinning of the CKO optic nerves (RGC axon bundles) appeared to temporally follow RGC degeneration (Fig. 2D). Although there was no difference in the thickness of the optic nerve at 1 month (Fig. 2E), CKO optic nerves at 9 months were significantly thinner compared with those of the controls (Fig. 2F). As observed in FD patients (Mendoza-Santiesteban et al., 2017), intrinsically photosensitive, melanopsin^+^ RGCs were preserved even at 18 months, despite extensive degeneration of conventional RGCs (Fig. 2G,H). This observation suggests that loss of IKAP differentially affects RGC subtypes.

**Fig. 3.**
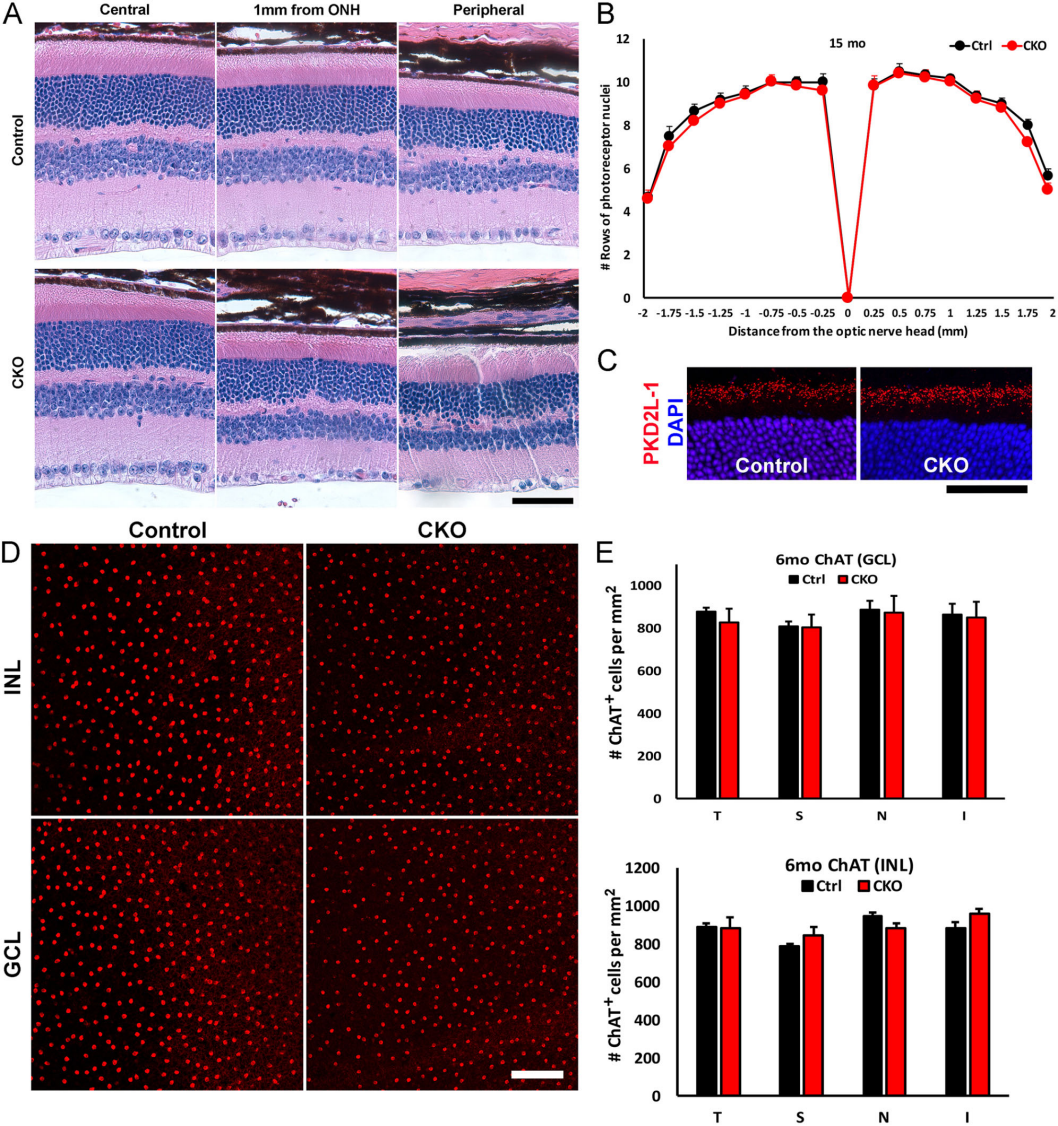
**Loss of IKAP does not cause degeneration of retinal neurons other than RGCs.** (A) H&E staining of 15 month retinal cross-sections at central (0.5 mm from the optic nerve head, ONH), middle (1 mm from ONH) and peripheral (1.5 mm from ONH) retina. Central retinas, where *Pax6-Cre* is not expressed, do not show any morphological differences between control and CKO mice. In the middle-peripheral retinas of CKO mice, where *Pax6-Cre* is active, reduction of RGCs in the ganglion cell layer (GCL) is apparent, whereas other cell types of the retina do not display any abnormal number and morphology. (B) The number of rows of photoreceptor nuclei in the outer nuclear layer was counted at 0.25 mm from the ONH in the temporal and nasal retinas at 15 months. There was no difference in the photoreceptor number between control and CKO retinas. The number was compared at the same distance with Student's *t*-test (*n*=6 for control; *n*=5 for CKO). For all points, *P*>0.05 (not significant). (C) IHC of cilia marker PKD2L-1(red) shows no abnormality in photoreceptor cilia structure between the inner segment and outer segment at 9 months. (D,E) The number of ChAT^+^ cholinergic amacrine cells in 6 month retinas was counted at 1 mm from the ONH in each quadrant. Representative images of the temporal inner nuclear layer (INL) and GCL ChAT^+^ cells (red) are shown (D). There was no difference in the number of ChAT^+^ cholinergic amacrine cells in either INL or GCL (E). *P*>0.05 with Student's *t*-test for all points (*n*=5). T, temporal; S, superior; N, nasal; I, inferior. Scale bars: 50 µm (A,C) and 100 µm (D).

We have previously shown that IKAP is expressed in RGCs, photoreceptors, amacrine cells and a subset of bipolar cells in adult mouse retinas (Ueki et al., 2016). Although the loss of RGCs was significant by 1 month in the CKO retinas (Fig. 2), histological analyses of 1, 3, 6, 9, 12 and 15 month CKO retinas did not show any additional retinal phenotype nor were any other retinal cell types affected by IKAP loss (Fig. 3 and Fig. S2). Immunohistochemistry (IHC) with various cell-type markers showed no significant difference in photoreceptor (Otx2), bipolar cell (Otx2), amacrine cell (Pax6) or Müller glial (Sox9, Sox2, GFAP) number, morphology or organization at 9 months (Fig. S2A). There was no abnormal morphology in the CKO retinas at 12 months compared with the controls (Fig. S2B). At 15 months, although retinal cross-sections of the Cre-affected area (middle-peripheral) show decreased RGC number, the overall retinal morphology remains intact (Fig. 3A and Fig. S2C). When the number of rows of photoreceptors were counted at 0.25 mm intervals from the optic nerve head, there was no difference in the number of photoreceptors between control and CKO retinas (Fig. 3B). In our previous study, *Ikbkap^−/−^* CNS neurons showed disrupted cilia morphology (Chaverra et al., 2017). Therefore, we analyzed photoreceptor cilia, located in between inner and outer segments. A cilia marker, PKD2L-1(red), showed no abnormality in photoreceptor cilia structure in CKO photoreceptors at 9 months, further indicating that photoreceptors are not affected by the absence of *Ikbkap*. As we also observed loss of cholinergic neurons in our other FD model (Chaverra et al., 2017), the number of ChAT^+^ cholinergic amacrine cells was counted in both the inner nuclear layer (INL) and ganglion cell layer (GCL) at 6 months (Fig. 3D,E). However, we did not observe any reduction in ChAT^+^ amacrine cells in the CKO retina. In summary, we determined that RGCs are the only cell type to require IKAP for their survival, even though *Pax6-Cre* is expressed in all retinal cell types.

### Mitochondrial membrane integrity and function is impaired in the absence of IKAP

**Fig. 4.**
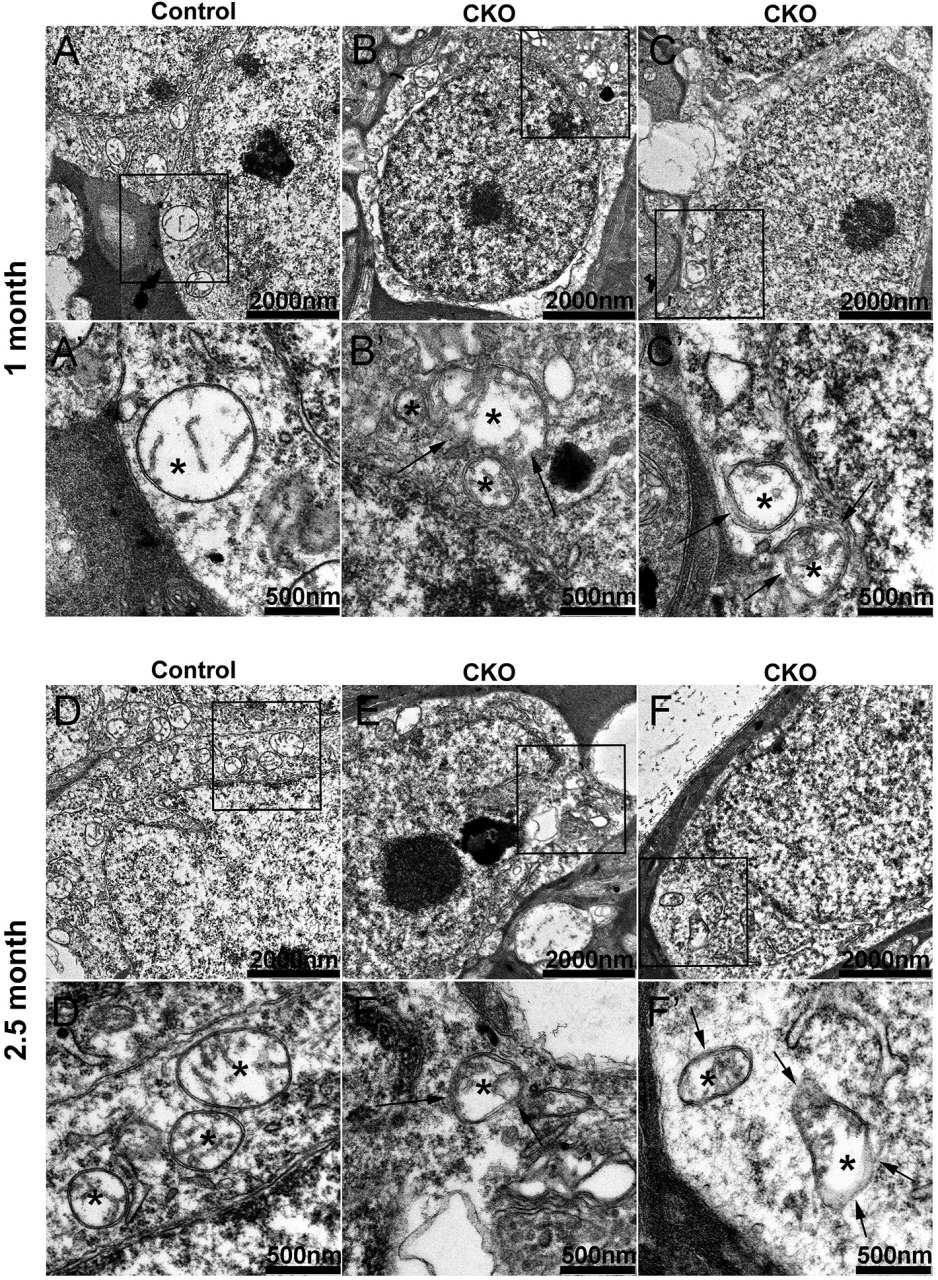
**Integrity of the mitochondrial membrane was lost in CKO RGCs.** Mitochondria of RGCs were visualized using TEM at (A-C) 1 month and (D-F) 2.5 months. At both 1 and 2.5 months, CKO mitochondria (B,C,E,F) show a disrupted double-membrane structure (arrows) compared with controls (A,D), indicating that loss of IKAP led to morphological impairment of mitochondria in RGCs. Representative images are shown. Asterisks indicate mitochondria. Squared regions in A-F are shown enlarged in A′-F′.

Our recent study revealed that mitochondria of embryonic *Ikbkap* CKO dorsal root ganglia neurons are depolarized, produce elevated levels of reactive oxygen species (ROS), are fragmented, and do not aggregate normally at axonal branch points (Ohlen et al., 2017). In support of a mitochondrial deficit in FD, FD patients have a temporal optic nerve degeneration that is reminiscent of LHON and DOA, both of which are caused by mutations that affect mitochondrial function (Carelli et al., 2009; Chevrollier et al., 2008; Kirches, 2011; Newman and Biousse, 2004; Yu-Wai-Man et al., 2011; Zanna et al., 2008). Therefore, we investigated the health of mitochondria in our CKO retinas. We performed transmission electron microscopy (TEM) on control and CKO retinas at 1 month and 2.5 months (Fig. 4 and Fig. S3). We observed a breakdown in the mitochondrial double-membrane integrity in CKO RGCs as early as 1 month, which is during the period of rapid RGC degeneration (Fig. 4). By contrast, although mitochondria of 2.5 month CKO amacrine cells show a similar loss of membrane integrity, mitochondria of 1 month CKO amacrine cells display no difference compared with controls (Fig. S3). Moreover, despite this slower progressive loss of membrane integrity, amacrine cells do not die in the absence of IKAP (Fig. S2A and Fig. 3D,E). This finding suggests that loss of IKAP affects different cell types at different rates, and that RGCs are more vulnerable to the loss of IKAP than are other retinal cell types.

**Fig. 5.**
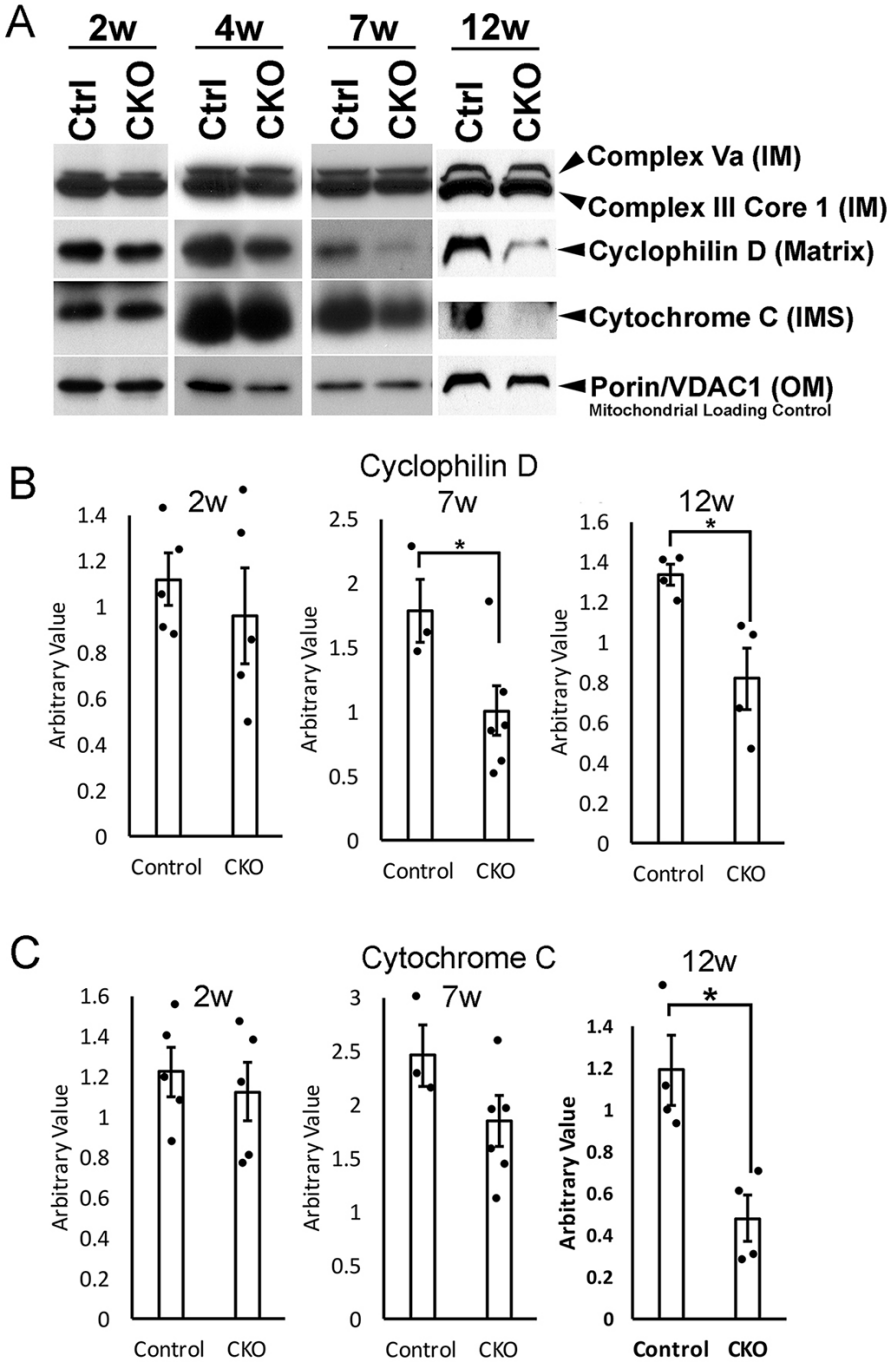
**Progressive loss of mitochondrial membrane integrity in the CKO retinas.** Retinas of the indicated ages (2, 4, 7 and 12 weeks) were collected and western blot analysis performed using the mitochondrial membrane integrity antibody cocktail. The cocktail included protein markers of the four mitochondrial compartments. (A) Representative blot. There was no difference in the expression of protein markers of the four mitochondrial compartments at 2 weeks (P14, at the completion of retinal development) between control and CKO retinas. A significant reduction in cyclophilin D and cytochrome *c* in CKO retinas was detected as the retina ages, suggesting progressive disruption in mitochondrial structure. IM, inner membrane; OM, outer membrane; IMS, intermembrane space. Equal amounts of retinal lysate proteins were loaded. (B) Western blot quantitation for cyclophilin D. There was a progressive decrease in cyclophlin D and a significant decrease in its expression was detected in CKO retinas at 7 weeks. *P<0.05 with Student's *t*-test; 2 weeks (*P*=0.26; *n*=5), 7 weeks (*P*=0.031; *n*=3 for control; *n*=6 for CKO) and 12 weeks (*P*=0.018; *n*=4). (C) Significant reduction in cytochrome *c* expression was observed in CKO retinas compared with controls by 12 weeks. *P<0.05 with Student's *t*-test; 2 weeks (*P*=0.31; *n*=5), 7 weeks (*P*=0.080; *n*=3 for control; *n*=6 for CKO) and 12 weeks (*P*=0.016; *n*=4).

Given the morphological disruption to mitochondrial membrane integrity detected in TEM, we sought to characterize membrane integrity at the molecular level. We collected retinas at 3 months and used an antibody cocktail that measures levels of mitochondrial proteins in four different mitochondrial compartments (outer and inner membrane, intermembrane space and matrix) (Fig. 5). Significant decreases in the mitochondrial proteins cyclophilin D and cytochrome *c* were detected in CKO retinas at 3 months (12 weeks), supporting the loss of membrane integrity seen in TEM analyses (Fig. 4 and Fig. S3). Loss of mitochondrial integrity in the CKO retinas was progressive: at 2 weeks (P14), the time when retinal development is completed, there was no difference in cyclophilin D and cytochrome *c* expression, but both proteins did progressively decline in expression in older CKO retinas (Fig. 5B,C). These data suggest that loss of *Ikbkap* during development does not affect retinal cell mitochondria. Although RGCs showed morphological mitochondrial abnormality and cell death as early as 1 month (Figs 2B and 4), we did not see a significant difference in the mitochondrial protein levels by western blot at 1 month (4 weeks). This is probably because RGCs only represent 0.5% of the total retinal population (Jeon et al., 1998) so that RGC changes are not reflected in the total retinal lysates. As retinas aged, however, we did measure a significant difference in whole retinal lysates after 7-12 weeks. This decrease in mitochondrial proteins in CKO retinas corresponds well with the morphological changes in mitochondria observed in other cell types of the retina (Fig. S3). Our western blot analysis suggests that the number of total mitochondria in the CKO retinas was unchanged, as the expression of mitochondrial loading control porin/voltage-dependent anion-selective channel protein 1 (VDAC1) was the same between control and CKO retinas (Fig. 5A). In support of this, IHC analysis of the RGC layer shows comparable levels of VDAC expression between control and CKO retinas at 3 months (Fig. S4), also suggesting that the total retinal mitochondrial number is unaffected by the loss of IKAP.

**Fig. 6.**
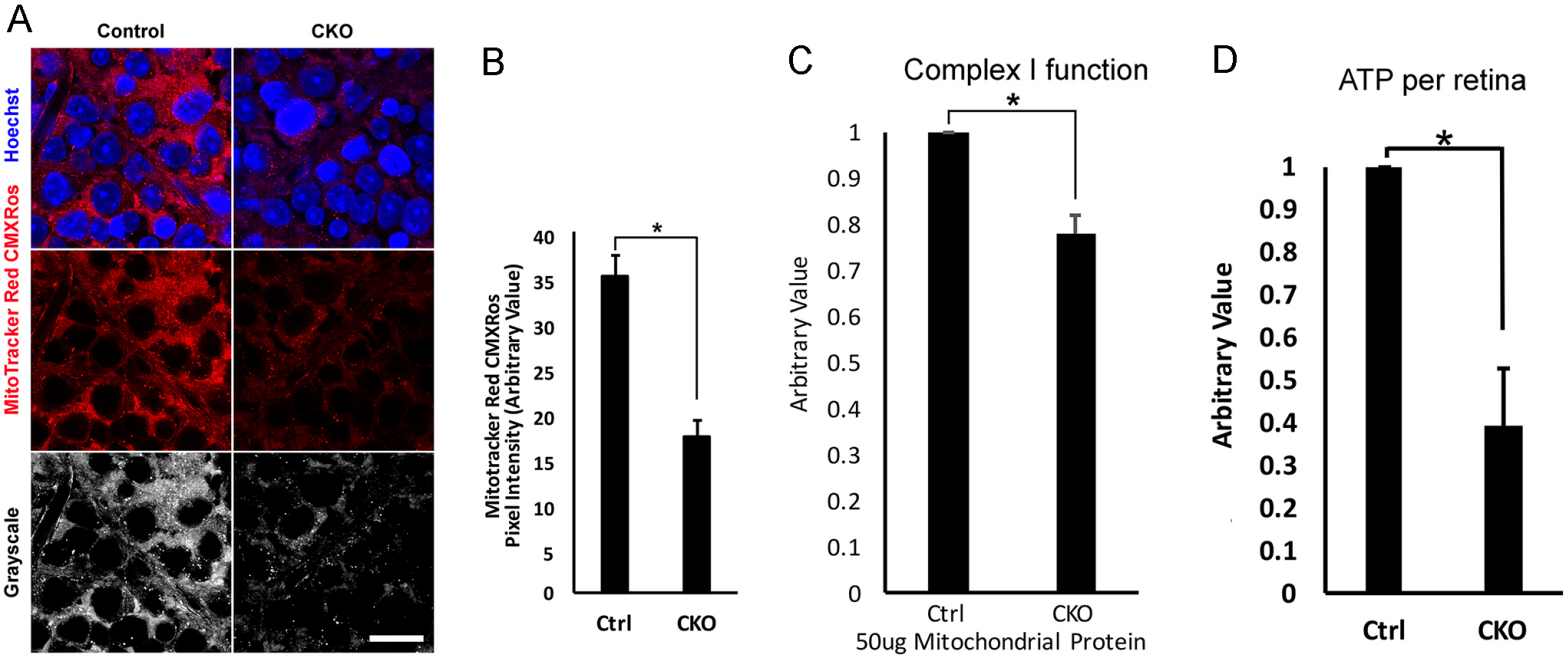
**Loss of IKAP causes mitochondrial membrane depolarization, impairs complex I function and decreases cellular ATP levels.** (A) Freshly isolated 3-month old retinas were treated briefly with MitoTracker Red CMXRos and Hoechst (nuclear marker), fixed and flatmounted for confocal imaging. Representative images of the RGC layer are shown. The signal intensity of MitoTracker Red CMXRos correlates with healthy mitochondrial membrane potential. Scale bar: 50 µm. (B) ImageJ analysis revealed a 50% decrease in MitoTracker Red CMXRos signal intensity in the CKO RGC layer compared with controls, indicating that there was significant loss of mitochondrial membrane potential in the cells of the RGC layer in CKO mice. *P=0.00026 with Student's *t*-test (*n*=5). (C) Mitochondria of 2.5 month control and CKO retinas were isolated and mitochondrial complex I function was measured. CKO retinas had a 22% reduction in complex I activity compared with controls. *P=0.0025 with Student's *t*-test (*n*=5). (D) Total ATP of the control and CKO retinas was measured from the total retinal lysates at 2.5 months. Total ATP content of the CKO retina was 40% that of the control. *P=0.0053 with Student's *t*-test (*n*=5).

To assess changes in the mitochondrial membrane potential, we briefly treated freshly isolated 3 month retinal explants with MitoTracker Red CMXRos, a dye retained by healthy mitochondria that have a normal membrane potential. MitoTracker images of the CKO RGC layer show a dramatic reduction in fluorescence signal (Fig. 6A,B), indicating a significant loss of mitochondrial membrane potential in these cells. To identify the mechanisms underlying the mitochondrial depolarization, we measured mitochondrial complex I function at 2.5 months. Complex I was chosen because its activity was decreased significantly in other mitochondrial optic neuropathies, such as LHON (Brown et al., 2000; Carelli et al., 1997). We measured a 22% decrease in complex I function in the CKO retinal mitochondria compared with controls (Fig. 6C). This would be an underestimate of complex I reduction in an affected CKO cell, as the central half of the CKO retinas were not affected by *Pax6-Cre*-mediated recombination of *Ikbkap* and were essentially the same as control retinas (Fig. 1). In support of this loss in complex I activity, the total cellular ATP concentration per retina was significantly decreased (60% decrease) in CKO retinas compared with controls (Fig. 6D). As most cellular ATP is generated in mitochondria, the results indicate impaired mitochondrial function in the CKO retinas. In summary, we demonstrate that there are significant reductions in mitochondrial integrity, membrane potential and function in the retina in the absence of IKAP.

## DISCUSSION

FD is caused by a point-mutation in *IKBKAP/ELP1*, a gene that encodes an elongator complex subunit, IKAP/ELP1. Although FD patients suffer from many congenital and progressive neuropathies, progressive blindness owing to the loss of RGCs is one of the most debilitating phenotypes suffered by patients as they age. To determine whether autonomous loss of IKAP function in the retina would recapitulate the human FD optic neuropathy, we generated a new mouse model in which the targeted deletion of *Ikbkap* was restricted solely to the retina with Cre expression in all retinal cell types (Fig. 1) (Marquardt et al., 2001). *Pax6-Cre* was able to abolish 70% of IKAP expression in the retina (Fig. 1). Interestingly, *Ikbkap* CKO retinas showed normal retinal development but demonstrated progressive RGC degeneration and optic nerve atrophy starting at 1 month of age (Fig. 2), whereas the survival of other retinal neurons was unaffected (Fig. 3). These findings indicate that not only is IKAP not required for retinal development, but in adulthood only RGCs, and not other retinal cell types, depend on its function for survival. Finally, we demonstrate here that *Ikbkap*-deficient retinal neurons, including RGCs, exhibit a loss of mitochondrial integrity and function and decreased levels of ATP (Figs 4-6), suggesting that mitochondrial dysfunction is one of the causes of RGC death in FD.

The retinal phenotype observed in our CKO mice recapitulates the human FD pathology. Although in the PNS, IKAP deficiency clearly affects neuronal development (Abashidze et al., 2014; George et al., 2013; Hunnicutt et al., 2012; Jackson et al., 2014), we did not observe any developmental defects in the CKO retinas. This finding suggests that PNS and CNS neurons have different susceptibility to the loss of IKAP, and that there are clearly two distinct stages of neuronal loss in the disease: developmental versus adult progressive degeneration. Analysis of the vision of FD patients revealed that loss of the RGCs and optic nerve fiber, especially in the maculopapillary region (temporal retina), is the cause of the progressive blindness in FD (Mendoza-Santiesteban et al., 2014, 2017). As also seen in our CKO mouse retinas, survival of retinal neurons other than RGCs is not compromised in the retinas of FD patients. Interestingly, a recent report on postmortem human FD retinas showed degenerating mitochondria in the temporal portion of the optic nerve head pre-laminar region (Mendoza-Santiesteban et al., 2017). This observation corresponds well with our finding that mitochondrial morphology and function are impaired in retinal neurons in the absence of *Ikbkap* (Figs 4-6). In addition, RGC degeneration was subtype-specific, with melanopsin^+^ RGCs being resistant to death even in the absence of *Ikbkap* (Fig. 2), confirming our previous finding in a mouse model in which *Ikbkap* was deleted from the majority of the CNS and PNS (Ueki et al., 2016). Melanopsin^+^ RGCs are also preserved in the retinas of human FD patients (Mendoza-Santiesteban et al., 2017). Our previous analysis conducted in an FD mouse model, in which *Ikbkap* was deleted in the CNS and PNS using a *Tuba1α-Cre*, also showed progressive RGC degeneration (Chaverra et al., 2017; Ueki et al., 2016). With that model, however, we were unable to ascertain whether the demise of RGC was the direct or indirect consequence of loss of RGC *Ikbkap*, given that the *Tuba1α-Cre Ikbkap* CKO model exhibited widespread PNS and CNS neurodegeneration, which could indirectly affect the integrity of the neural retina (Chaverra et al., 2017; Ueki et al., 2016). To overcome these complications, we generated a retina-specific *Ikbkap* CKO and demonstrate here that RGCs autonomously require *Ikbkap* for their survival. In summary, our retina-specific *Ikbkap* CKO is an excellent FD model to study mechanisms of RGC degeneration and to test potential therapeutics in otherwise healthy mice.

Why neurons die in the absence of IKAP is not fully resolved, nor have we thoroughly identified its function(s) in neurons. In other systems, loss of IKAP also impairs target innervation, exocytosis, cytoskeletal organization and axonal transport, which might directly and/or indirectly lead to cell death (Abashidze et al., 2014; Chaverra et al., 2017; Close et al., 2006; George et al., 2013; Hunnicutt et al., 2012; Jackson et al., 2014; Johansen et al., 2008; Lefler et al., 2015; Naftelberg et al., 2016; Naumanen et al., 2008; Tourtellotte, 2016). IKAP/ELP1 is the scaffolding subunit of the six-subunit elongator complex, which is required for the translation of codon-biased genes. The wobble uridine (U_34_) of tRNAs that recognize both AA- and AG-ending codons is modified by the addition of both a thiol (s^2^) and a methoxy-carbonyl-methyl (mcm^5^). This double modification enhances the translational efficiency of AA-ending codons (Bauer and Hermand, 2012; Chen et al., 2009b; Huang et al., 2005). Importantly, these specific wobble uridine modifications in tRNA are reduced in FD patients (Karlsborn et al., 2014) and FD mice (Goffena et al., 2018). One important class of mRNAs that we discovered were codon-biased, and hence their proteins were expressed at lower levels in *Ikbkap* CKO peripheral neurons, are those that repair DNA damage (Goffena et al., 2018). Interestingly, mitochondrial DNA can also be damaged in neurodegenerative disease (Van Houten et al., 2016) and, if unrepaired, can lead to an elevation in ROS and trigger apoptosis. Furthermore, in the developing PNS, we have shown that *Ikbkap^−/−^* neurons die through a p53/activated caspase-3-mediated apoptosis (George et al., 2013). Whether this death results from the direct or indirect effects of impaired tRNA modification is still unresolved, however. Finally, a study in yeast shows that wobble uridine modification by the elongator complex (Elp1-6) is required for mitochondrial function under stress conditions (Karlsborn et al., 2014; Tigano et al., 2015). Thus, although it is clear that the loss of IKAP and elongator function cause intracellular stress, including severe impairments in mitochondrial morphology and function, elucidating the exact cellular and molecular pathways connecting the loss of elongator function to mitochondrial damage and neuronal death in FD requires future study.

Recently, we demonstrated that mitochondria of *Ikbkap* CKO embryonic PNS neurons were fragmented, had disrupted membrane potential and had increased ROS (Ohlen et al., 2017). In this new study, we show that mitochondria of *Ikbkap*-deficient retinal neurons are also morphologically and functionally impaired (Figs 4-6), demonstrating that mitochondrial dysfunction occurs in both developing and adult neurons in the PNS and CNS in the absence of IKAP. Our TEM analysis revealed disruption of mitochondrial membranes in *Ikbkap*-deficient retinal neurons (Fig. 4 and Fig. S3). Mitochondrial translocation of the p53 tumor suppressor protein has been shown to disrupt inner and outer mitochondrial membrane integrity (Wolff et al., 2008). In line with this, given the elevated p53 activity in PNS neurons (George et al., 2013), it is possible that p53 might contribute to the breakdown in mitochondrial membrane integrity in *Ikbkap* CKO retinal neurons.

Furthermore, we demonstrate here that *Ikbkap* CKO retinas had decreased complex I activity and ATP levels compared with control retinas, which might explain the poor growth, rhabdomyolysis and reduced muscle tone observed in FD patients (Axelrod, 2002; Riley et al., 1949). Intriguingly, a muscle biopsy conducted on an FD patient revealed a severe impairment of mitochondrial complex I, III and IV activity (A.S., personal communication). Decreased ATP levels have also been detected in the optic nerve of a mouse glaucoma model, which had experienced RGC degeneration owing to elevated intraocular pressure (Baltan et al., 2010). Interestingly, reduced levels of ATP are a common hallmark of the major neurodegenerative disorders, including Alzheimer's disease, Parkinson's disease and ALS (Pathak et al., 2013). Although total mitochondrial protein levels were not reduced in the *Ikbkap* CKO retina, ATP levels were reduced by 60% which is commensurate with the 70% loss of IKAP protein. What is surprising, however, is that despite Cre being expressed in approximately 90% of retinal cells in the peripheral retina, only 40-60% of the RGCs die and none of the other retinal cell types die. This is similar to observations made in the embryonic dorsal root ganglia where approximately half of the tropomyosin receptor kinase A (TrkA^+^) nociceptors and thermoreceptor subpopulation die in the absence of IKAP, yet the TrkC^+^ subset (which comprise the proprioceptors) do not die. Thus, for reasons we do not currently understand, different neuronal populations have a differential dependency on the IKAP protein for their survival. Although these resilient neurons can survive, they might not be as healthy as *Ikbkap^+/+^* cells, as evidenced by the breached mitochondrial morphology in several amacrine cells (Fig. S3 and Fig. 3D,E); thus, these neurons might produce less ATP, which explains the 60% reduction in ATP levels observed in the CKO retina. Given the normal cellular variability in ATP production, our measurement of total retinal ATP content would not accurately reflect any changes in ATP concentration per single cell. What is intriguing, however, is that recent work has demonstrated that, although ATP concentrations in a healthy cell are in the millimolar range, only micromolar concentrations of ATP are required for bioenergetics. By contrast, an intracellular concentration of ATP that is 1000-fold higher than required enables it to function as a hydrotrope to keep proteins in solution and dissolve protein aggregates that form within the cytosol (Patel et al., 2017). As protein aggregation is a common hallmark of all major neurodegenerative diseases, and has been shown to occur in other elongator mutant systems (Laguesse et al., 2015), it is conceivable that the reduction in ATP we demonstrate in *Ikbkap^−/−^* neurons might exacerbate intracellular stress by triggering protein aggregation. Hence, these reduced ATP levels might contribute to the progressive degeneration of several cell types in FD as patients age, a topic to be pursued in future studies.

Two retinal disorders, LHON and DOA, and FD share remarkable similarity in their disease progression: all are characterized by loss of vision owing to slow, progressive RGC degeneration (Fig. 2) (Carelli et al., 2009; Chevrollier et al., 2008; Kirches, 2011; Mendoza-Santiesteban et al., 2017; Newman and Biousse, 2004; Ueki et al., 2016; Yu-Wai-Man et al., 2011; Zanna et al., 2008). Both LHON and DOA are mitochondrial diseases: the genes that are mutated function in mitochondria. LHON is generally caused by a point-mutation in one of three mitochondrial genes, all three of which encode mitochondrial complex I subunits (Howell et al., 1991; Johns et al., 1992; Wallace et al., 1988); the LHON retina has decreased complex I activity and reduced ATP synthesis that results in progressive RGC degeneration. In DOA, mitochondrial fusion is impaired. Interestingly, in both LHON and DON, melanopsin-containing RGCs are spared, despite the extensive degeneration of conventional RGCs (La Morgia et al., 2011, 2010; Moura et al., 2013) as seen in FD mouse retinas (Fig. 2) (Ueki et al., 2016) and FD patients (Mendoza-Santiesteban et al., 2017). Mitochondrial dysfunction is also implicated in glaucoma, which is characterized by RGC death (Kim et al., 2015; Osborne and del Olmo-Aguado, 2013). Together, we conclude that mitochondrial dysfunction is one of the causes of RGC degeneration in FD.

To our surprise, despite their mitochondrial integrity and function also being impaired due to loss of *Ikbkap*, cell types other than RGCs were spared from death in the CKO retina (Fig. 2). Our TEM analysis revealed that the mitochondrial membrane integrity of amacrine cells was also progressively disrupted, but at a reduced pace and scale compared with that of RGCs (Fig. S3). Significant reduction in mitochondrial membrane proteins, complex I function and ATP content was observed from total retinal lysates or mitochondria of CKOs, compared with controls (Figs 5 and 6). This observation suggests that mitochondria of all retinal cells that express Cre (50-85%) in the CKO retina (Fig. 1) are affected, as RGCs only account for 0.5% of retinal cell populations (Jeon et al., 1998). In support of this, mutations in the mitochondrial gene/protein in LHON and DOA also cause degeneration of RGCs, but not of the other retinal neurons (Yu-Wai-Man et al., 2011). Why RGCs of all retinal cell types are the most vulnerable to loss of *Ikbkap* is unknown. One explanation is that the RGCs have a high energy demand and are vulnerable to mitochondrial dysfunction owing to their unique anatomy: RGC axons are only myelinated beyond the lamina cribosa (where the optic nerve starts), and therefore propagation of action potentials is less efficient and requires more energy in the unmyelinated prelaminar region of the RGC axons (Morgan, 2004). The brain consumes 20% of the basal metabolic rate (Clarke and Sokoloff, 1999), with the visual system being one of the most energy-consuming systems. Moreover, the retina is one of the tissues with the highest oxygen demand (Ames et al., 1992; Anderson and Saltzman, 1964; Niven and Laughlin, 2008). Cytochrome *c* oxidase (COX) is a terminal enzyme of the mitochondrial electron-transport chain and is necessary for ATP production (Wikstrom, 1977). Therefore, the level and activity of COX reflects energy demand. In retina, strong COX expression and activity is detected in the nerve fiber layer, the unmyelinated RGC axon bundle within the retina, and in the prelaminar and laminar regions of the optic nerve (Andrews et al., 1999). These unmyelinated regions of RGC axons make them especially vulnerable to energy deprivation (Andrews et al., 1999; Morgan, 2004), causing RGCs to degenerate under metabolic dysfunction. In fact, in FD, LHON and DOA patients, RGCs of the more metabolically taxed temporal retinas are the first to die, before pan-retinal RGC degeneration is observed (Mendoza-Santiesteban et al., 2014, 2017; Wakakura et al., 2009; Yu-Wai-Man et al., 2014). In addition, among all the retinal neurons, IKAP expression is highest in the RGCs (Ueki et al., 2016), suggesting that RGCs might be more dependent on normal IKAP function than other retinal neurons. In summary, our data clearly indicate that RGCs are the cell type to target with potential treatments for FD blindness.

Whether the loss of mitochondrial integrity and function observed in our CKO retinas is a direct cause of RGC death or not remains currently unresolved. However, our work on *Ikbkap*-deficient dorsal root ganglia (DRG) neurons demonstrates that improvement of mitochondrial function in FD DRG neurons leads to increased neuronal survival (Ohlen et al., 2017), suggesting that mitochondria are promising targets for preventing RGC loss in FD. Given the remarkably similar phenotypes between FD, LHON and DOA, understanding the mechanisms of RGC loss in FD, and identifying potential therapeutics, might help mitigate progressive RGC loss not only in FD, but also in other mitochondrial optic neuropathies.

## MATERIALS AND METHODS

### Mice

All mice were housed in the Animal Resource Center at Montana State University and protocols were approved by the Montana State University Institutional Animal Care and Use Committee. Retina-specific *Ikbkap* CKO mice were generated by crossing *Ikbkap* floxed (International Knockout Mouse Consortium) and *αPax6* promoter-driven Cre (*Pax6-Cre*) mice (Marquardt et al., 2001), which were a gift from the original founders (Drs Peter Gruss and Ruth Ashery-Padan). *Pax6-Cre* is expressed in the retinal progenitors, allowing *Ikbkap* deletion from all the retinal neurons and Müller glia (Marquardt et al., 2001). Both male and female mice were used at the age indicated, and littermate *Pax6-Cre^−^;Ikbkap^flox/flox^* mice were used as controls. To analyze *Cre* expression in the retina and to determine affected retinal regions, *Pax6-Cre* mice were crossed to *Rosa-EGFP Cre* reporter mice (Jackson Laboratory, stock #004077).

### Western blots

Retinas were collected at the age indicated and standard western blot procedures were performed. An equal amount of total protein was loaded in each well. Primary antibodies used were as detailed: anti-IKAP (1:2000; Anaspec #AS-54494), mitochondrial membrane integrity cocktail (1:1000; Abcam #ab110414) and anti-β-actin (1:10,000; Santa Cruz Biotechnology #sc-47778). Quantitation of blots was performed using ImageJ and values were normalized using a loading control. Statistical analysis was performed using Student's *t*-test. Data are considered significant when *P*<0.05.

### Immunohistochemistry, RGC counts and H&E staining

Mice were euthanized with CO_2_ and the eyes were marked with a green tattoo dye on the temporal surface. IHC and confocal imaging were performed as described (Ueki et al. 2016). Primary antibodies used were as detailed: anti-GFP (1:5000; Abcam #ab13970), anti-Brn3 (1:250; Santa Cruz Biotechnology #sc-6026), anti-RBPMS (1:500; PhosphoSolutions #1832-RBPMS), anti-melanopsin (1:5000; Advanced Targeting Systems AB-N38), anti-PKD2L-1 (1:500; Millipore #AB9084) and anti-ChAT (1:200; Millipore #AB114P). For Brn3^+^ or melanopsin^+^ RGC counts on flatmounts, confocal images were taken at 1 mm from the optic nerve head in temporal, nasal, superior and inferior retinas, and the number of Brn3^+^ cells in each image was counted manually. The number of cells in 1 mm^2^ of the retina was calculated and plotted. The optic nerve was carefully cut away from the eyecup after fixation, rinsed in phosphate-buffered saline (PBS) and cryoprotected overnight in PBS plus 20% sucrose. Optic nerves were then embedded in OCT and cryostat-sectioned transversely at 16 μm. DAPI staining was performed to visualize the optic nerve, the circumference of each optic nerve was measured and plotted. Statistical analysis was performed using Student's *t*-test. Data are considered significant when *P*<0.05. Hematoxylin and Eosin (H&E) staining and photoreceptor counting were performed according to Ueki et al. (2016).

### Transmission electron microscopy

Eyes were collected and fixed in Karnovsky's fixative overnight at 4°C, washed in Ringer's saline and treated with 2% osmium tetroxide in 0.1 M potassium-sodium phosphate buffer (PSPB) for 4 h at room temperature. Eyes were rinsed three times with PSPB and the cornea and lens were carefully removed. The remaining eyecups were cut into four equal pieces under a dissecting microscope. Eyecups were dehydrated in a graded concentration series of ethanol (50-100%) and then in propylene oxide (PO). Infiltration of eyecups was performed in PO/Spurr's resin (2:1 vol:vol, 2:2 vol:vol, then 100% resin; overnight at 4°C for each). Each eyecup quarter was embedded in Spurr's resin and polymerized in a 70°C oven overnight. Sectioning was performed using an ultramicrotome with a diamond knife, and sections were collected and placed on mesh copper grids. Sections were then stained with uranyl acetate and lead citrate. TEM imaging was carried out using a LEO 912 (Zeiss) operating at 100 kV accelerating voltage. Sections from the peripheral retina were imaged and retinal cell types were identified by morphology, location and nuclear size (Jeon et al., 1998).

### Mitotracker treatment

Retinas were isolated and incubated with 200 nM MitoTracker Red CMXRos (Thermo Fisher Scientific) and Hoechst 33342 (Thermo Fisher Scientific) in neurobasal media at 37°C for 30 min. Chloromethyl X-rosamine (CMXRos) does not accumulate in depolarized mitochondria. Retinas were washed with PBS and fixed with 4% PFA for 5 min at room temperature. After fixation, retinas were washed and then flatmounted on a slide. Confocal imaging was performed immediately. Images (single focal plane) of the RGC layer at 1.75 mm from the optic nerve head were captured and the intensity of MitoTracker Red CMXRos quantified using ImageJ.

### Complex I activity

Retinas were pooled and gently lysed with a Dounce homogenizer (20-25 strokes). The mitochondrial fraction was isolated according to Dimauro et al. (2012). The concentration of the mitochondrial proteins was measured using a BCA protein assay kit (Thermo Fisher Scientific) and a 50 µg aliquot of mitochondrial protein was used for the assay. Mitochondrial complex I activity was measured using Complex I Enzyme Activity Microplate Assay Kit (Abcam). Each experiment was run in duplicate. The amount of complex I activity in CKO retinas was plotted compared with control retinas. Statistical analysis was performed using Student's *t*-test.

### ATP assay

The amount of ATP was measured using a StayBrite Highly Stable ATP Bioluminescence Assay Kit (BioVision). Each experiment was run in duplicate. ATP per retina was calculated and plotted compared with control retinas. Statistical analysis was performed using Student's *t*-test.

## Supplementary Material

Supplementary information
