# Supplementary material for: Retina-specific loss of Ikbkap/Elp1 causes mitochondrial dysfunction that leads to selective retinal ganglion cell degeneration in a mouse model of familial dysautonomia
Source: Dis Model Mech. 2018 Jul 30;11(7):dmm033746. doi: 10.1242/dmm.033746 (PMC6078410; doi:10.1242/dmm.033746)
Supplement: Supplementary information [file dmm-11-033746-s1.pdf]

SUPPLEMENTAL FIGURES

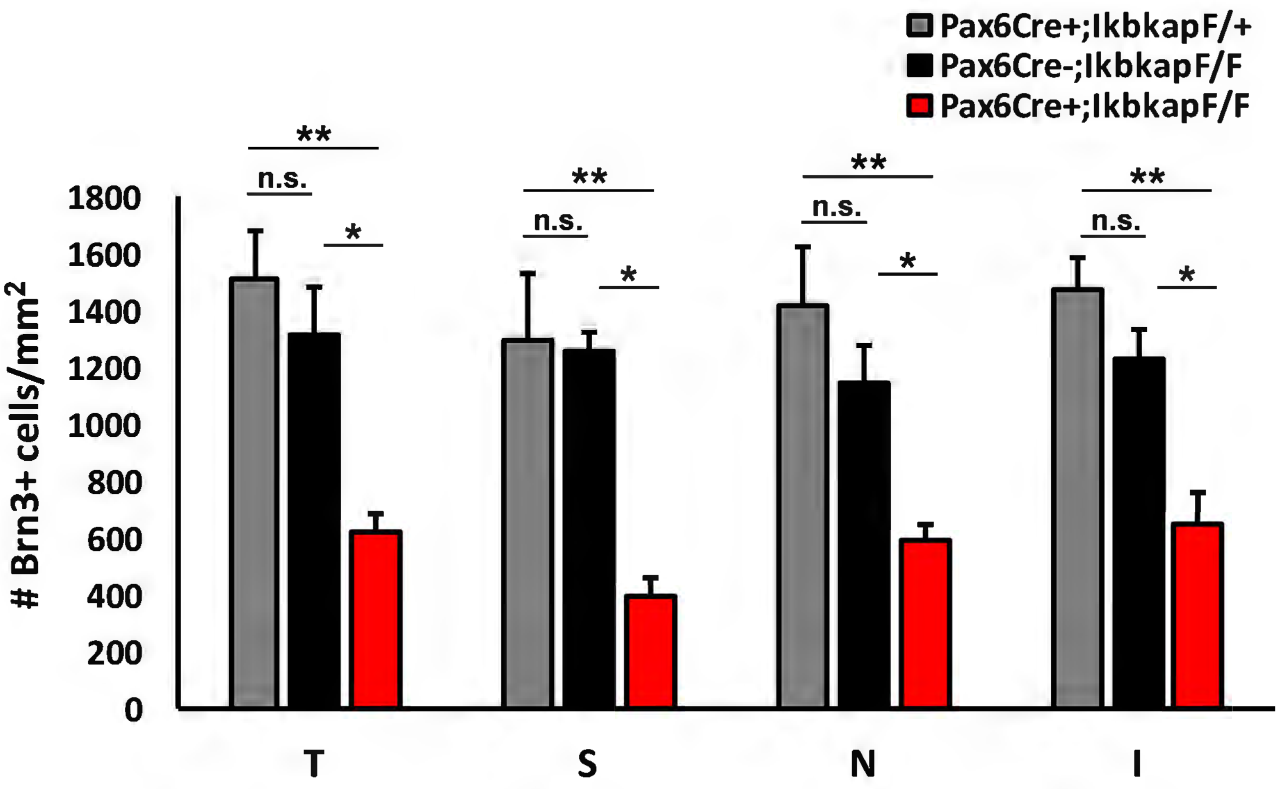

Figure S1 The number of Brn3$^+$ RGCs were counted in each quadrant of 18 month old *Pax6-Cre$^+$; Ikbkap$^{f/+}$*, *Pax6-Cre$^-$; Ikbkap$^{F/F}$* (control) and *Pax6Cre$^+$; Ikbkap$^{F/F}$* (CKO) retinas at 1mm from the ONH. There was no significant decrease in the number of RGCs in *Pax6-Cre$^+$; Ikbkap$^{f/+}$* compared to *Pax6-Cre$^-$; Ikbkap$^{F/F}$* retinas, demonstrating that Cre expression itself and/or loss of one *Ikbkap* allele did not cause RGC degeneration. *Pax6-Cre$^+$; Ikbkap$^{F/F}$* CKO retinas had significant reduction in the RGC number compared to *Pax6-Cre$^+$; Ikbkap$^{f/+}$ and Pax6-Cre$^-$; Ikbkap$^{F/F}$* retinas, showing that the loss of *Ikbkap* causes RGC degeneration. S, superior; N, nasal; I, inferior; T, temporal. Error bars represent SEM. *p<0.05 and **p<0.01 using ANOVA with post-hoc Tukey HSD test. n.s., not significant. n=5 for *Pax6-Cre$^+$; Ikbkap$^{f/+}$*, n=6 for *Pax6-Cre$^-$; Ikbkap$^{F/F}$* and n=5 for *Pax6-Cre$^+$; Ikbkap$^{F/F}$*.

Disease Models & Mechanisms • Supplementary information

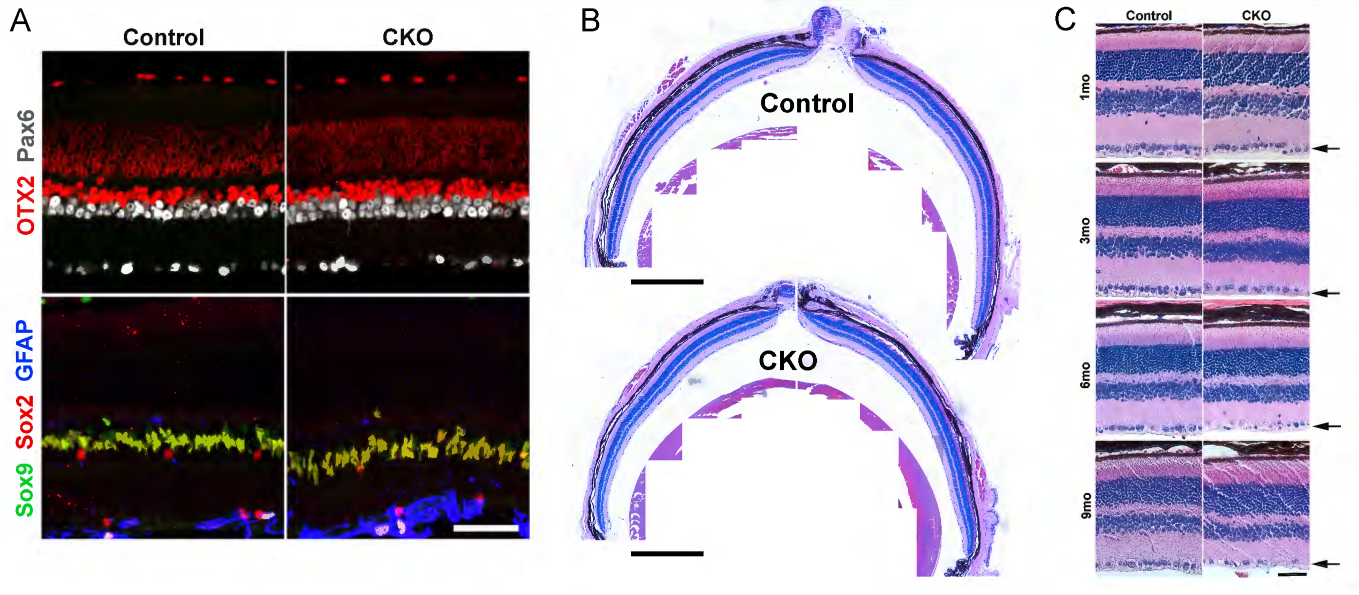

Figure S2 Overall retinal morphology is not affected by the loss of IKAP in CKO retinas. (A) Using several markers to visualize subtypes of retinal cells at 9 month revealed no apparent differences in control and CKO retinas. Pax6 (white), RGC, Müller glia, amacrine, and horizontal cell marker; OTX2 (red), photoreceptors, bipolar cells, and retinal pigment epithelial marker; GFAP (blue) and Sox9 (green), Müller glial marker; Sox2 (red), Müller glia and amacrine cell marker. (B) H&E staining of the 12 month control and CKO eyes show no apparent morphological differences. (C) Representative H&E staining of 1, 3, 6 and 9 month retinal cross sections at 1mm from the optic nerve head is shown. Reduction of RGCs in the GCL (arrows) is apparent while other retinal cell types do not display any abnormal number or morphology. Scale bars in (A,C) 50µm and (B) 0.5mm.

Disease Models & Mechanisms • Supplementary information

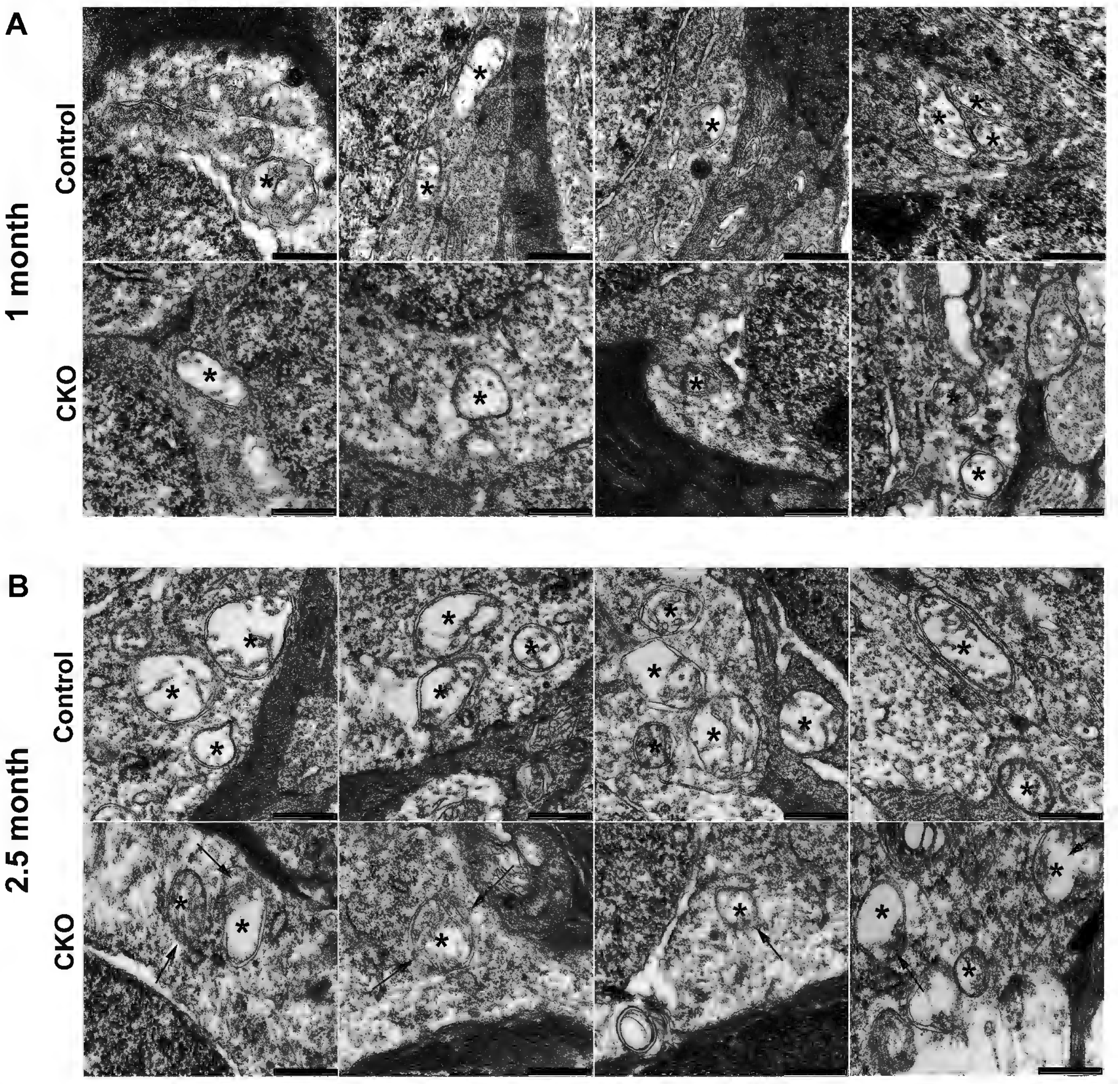

Figure S3 Mitochondria of CKO amacrine cells show loss of membrane integrity at 2.5 months but not at 1 month. Mitochondria of amacrine cells were visualized using transmission electron microscope (TEM) at 1month (A) and 2.5 month (B). Four representative amacrine cells are shown for each time point. Mitochondria are indicated with *. (A) At one month, there was no apparent difference in mitochondrial morphology between control (top) and CKO (bottom) amacrine cells. (B) At 2.5 months, disruption in mitochondrial double membrane structure was seen in CKO amacrine cells (arrows). Scale bars: 500nm.

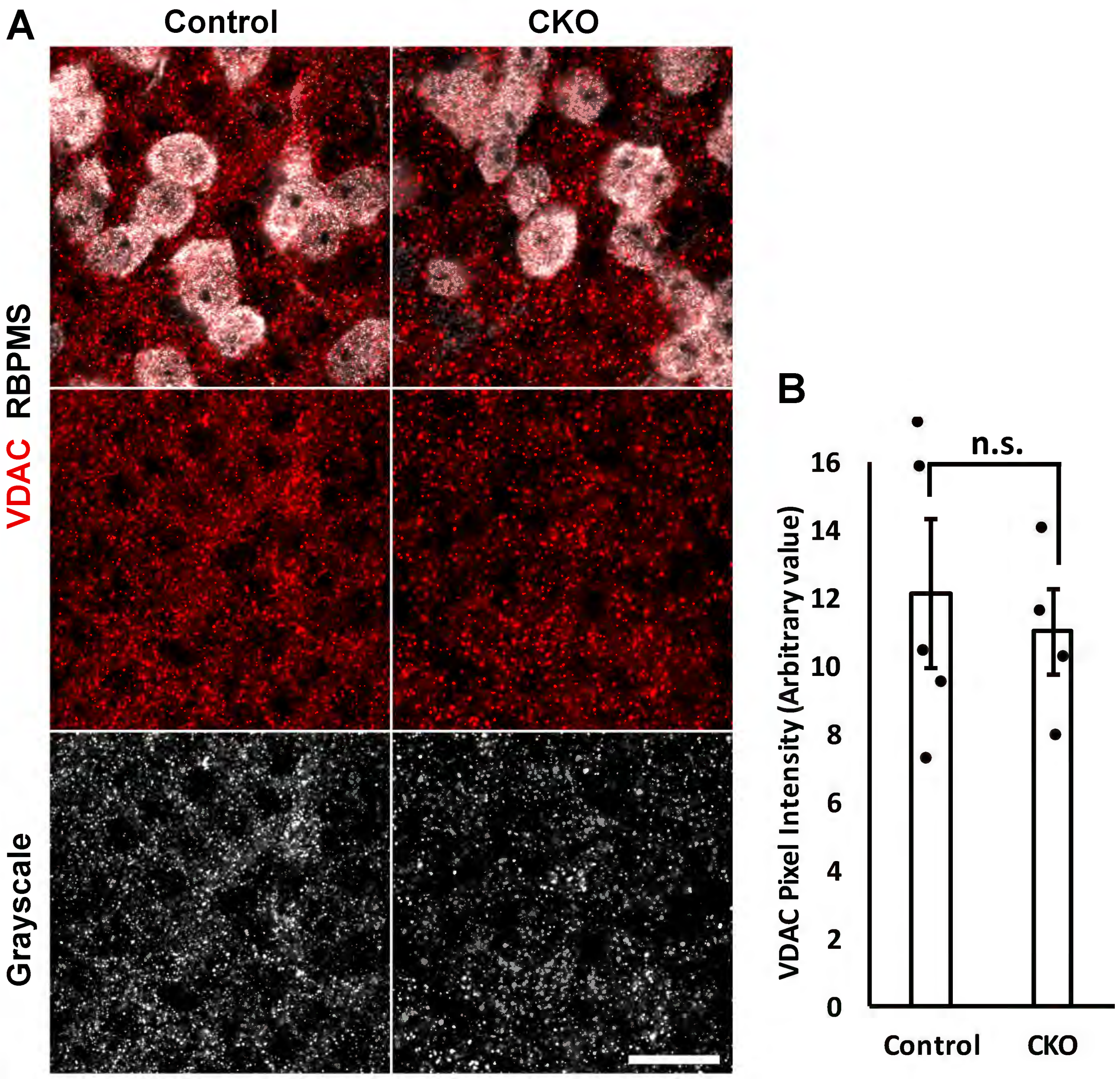

Figure S4 Loss of IKAP does not affect the total number of mitochondria in the CKO retinas. IHC for a pan-RGC marker RBPMS (white) and a mitochondrial marker VDAC (red and grayscale) was performed on 3 month retinal flatmounts. Scale bar: 50μm. (A) Representative images of the ganglion cell layer (GCL) are shown. (B) Pixel intensity of the VDAC signal was measured using image J. There was no difference in VDAC expression between control and CKO cells of the GCL, suggesting that the number of mitochondria in the GCL was unaffected by the loss of IKAP. n=5 for control and n=4 for CKO. p=0.34 with t-test (n.s., not significant).
